# Hot-Pressed Multicomponent Recycled Textile Polymer Blends Reinforced with Ground GFRP from Wind Turbine Blades: Microstructure–Property Relationships

**DOI:** 10.3390/ma19071306

**Published:** 2026-03-26

**Authors:** Maciej Wędrychowicz, Władysław Papacz, Janusz Walkowiak, Jagoda Kurowiak, Bartosz Siwczyk, Tomasz Skrzekut, Piotr Noga, Dominika Skarupska

**Affiliations:** 1 Institute of Materials and Biomedical Engineering, Faculty of Engineering and Technical Sciences, University of Zielona Gora, Prof. Z. Szafrana Street, 65-516 Zielona Gora, Poland; j.kurowiak@iimb.uz.zgora.pl (J.K.); 112649@stud.uz.zgora.pl (D.S.); 2 Institute of Mechanical Engineering, Faculty of Engineering and Technical Sciences, University of Zielona Gora, Prof. Z. Szafrana Street, 65-516 Zielona Gora, Poland; w.papacz@iim.uz.zgora.pl (W.P.); jwalkowiak@uz.zgora.pl (J.W.); b.siwczyk@iim.uz.zgora.pl (B.S.); 3 Faculty of Non-Ferrous Metals, AGH University of Science and Technology, A. Mickiewicza Av. 30, 30-059 Cracow, Poland; skrzekut@agh.edu.pl (T.S.); pionoga@agh.edu.pl (P.N.)

**Keywords:** recycled polymer blends, thermoplastic recycling, circular composites, short-fragment reinforcement, mechanical behavior, interfacial interactions, composite recycling

## Abstract

This study investigates hot-pressed composite plates manufactured from pellets obtained by mechanical recycling of post-consumer textile waste and reinforced with ground glass-fiber-reinforced polymer (GFRP) originating from wind turbine blades. Composite plates with dimensions of 200 × 330 × 8 mm were produced by hot pressing at 240 °C under 2 MPa with a heating and pressing time of 40 min. The recycled textile-derived polymer blend served as the matrix, while ground GFRP was introduced at 0, 10, 20, and 30 wt.%. Mechanical performance was evaluated using flexural and Charpy impact tests. The composites exhibited flexural strengths in the range of 9–13 MPa and impact strengths of 7.3–8.9 kJ m^−2^. The results did not reveal a monotonic increase in flexural strength with increasing reinforcement content. The highest average flexural strength was observed for the unreinforced matrix, while the addition of ground GFRP resulted in comparable or slightly lower strength values accompanied by increased scatter at higher reinforcement levels. The observed behaviour may be associated with heterogeneous dispersion of ground GFRP fragments, reduced effective reinforcement length due to mechanical grinding, interfacial constraints, and defect formation within the press-consolidated structure. The findings provide insight into the structure–property relationships of recycled composite systems based on heterogeneous textile-derived polymer blends.

## 1. Introduction

**Figure 1:**
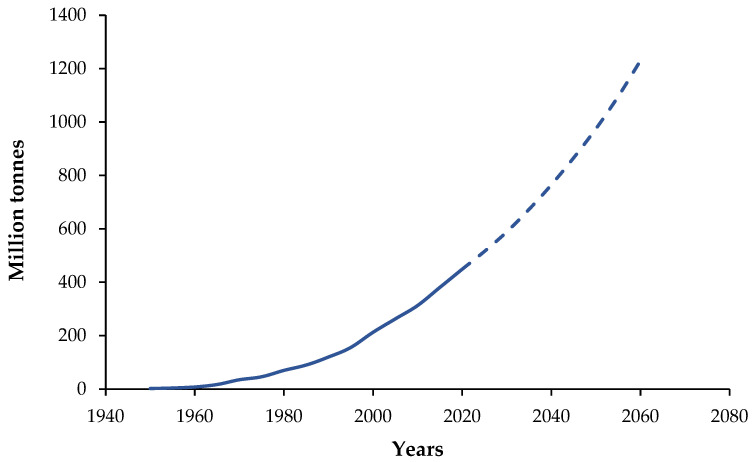
Historical global plastics production (solid line) and published projections to 2060 (dashed line; projection from 2020 onward). Data compiled from [1,2].

The global production of plastics has increased continuously since the mid-20th century and is projected to keep rising in the coming decades, leading to rapidly growing volumes of post-consumer polymer waste and increasing pressure on recycling systems (Figure 1). Projections reported in widely used datasets and policy outlooks indicate that global plastic production may continue to increase substantially up to 2060 under business-as-usual scenarios, highlighting the urgent need for scalable recycling and upcycling routes for polymer-based materials and products [1]. As a consequence, considerable research efforts are currently focused on developing technologies capable of transforming heterogeneous polymer waste streams into value-added materials with stable and predictable properties.

Although widely used thermoplastics are increasingly recycled, a key technological challenge remains the conversion of heterogeneous, multi-component waste streams into materials with stable and reproducible properties. General reviews emphasize that both mechanical and chemical recycling routes face limitations linked to feedstock variability and contamination in complex waste streams, as well as degradation accumulated during service life and reprocessing [3,4,5,6]. In practice, mechanical recycling dominates for many streams; however, repeated processing and use history can alter molecular weight distribution and rheology, and thus mechanical performance [7,8,9,10,11,12,13]. Consequently, robust upcycling concepts should account for degradation and variability instead of reporting mechanical results without context [8,9,10,14].

A representative example is post-consumer textile waste, which often results in recyclates containing mixtures of different polymers, additives, and fibrous fractions. Due to limited compatibility between the constituents, the sensitivity of certain components to moisture, and the presence of contaminants, microstructural defects such as porosity, inclusions, and weak interfacial bonding can readily form during melt processing. These defects have a significant impact on the mechanical performance of the material, particularly its stiffness, strength, and impact resistance [15,16]. Recent studies on composites derived from textile waste highlight that controlling morphology and improving interfacial quality—often through the use of compatibilization strategies—are essential to maintaining balanced mechanical properties at higher waste loadings [15].

Beyond individual case studies, reviews covering textile waste valorization highlight recurring bottlenecks relevant to engineering design: mixed polymer compositions, inconsistent feedstock, limited interfacial adhesion, and defect formation during processing; as a result, compatibilization strategies, tailored preprocessing and process-window control are frequently required to stabilize properties [15,16].

Recent studies have demonstrated that textile waste can be successfully incorporated into thermoplastic composites, including systems based on polypropylene, polyethylene, and polyamide matrices [15,16,17]. However, the mechanical properties of such materials often remain highly sensitive to feedstock variability and phase compatibility between different polymer fractions. As a result, improvements in composite performance typically require careful control of morphology, dispersion of reinforcing phases, and interfacial adhesion between the matrix and the reinforcement components [15,16,17,18].

These conclusions are consistent with broader findings for recycled polymer blends and polyamide, polyester containing recyclates, where mechanical recycling can significantly affect viscosity, crystallinity and property scatter [6,10,17,18]. Therefore, textile-derived recyclates should be treated as a polymer-blend feedstock whose performance is governed by both chemistry and microstructure, rather than as a single-polymer system [4,8,14,17].

In parallel, end-of-life glass-fiber-reinforced polymer (GFRP) waste is emerging as a major challenge due to increasing volumes, limited disposal options and the intrinsic complexity of cured composite architectures. Mechanical recycling (shredding and milling) is among the most industrially accessible approaches and generates particulate fractions that can potentially be reused as fillers or reinforcements in new materials, provided that dispersion and porosity are controlled. The literature on mechanically ground wind-turbine-blade waste illustrates both the scale of the problem and the practical relevance of microstructure control (in particular porosity) for property retention in secondary products [19]. Importantly, there is a growing body of work showing that milled ground GFRP can be integrated into thermoplastic matrices (including recycled ones), but the response is rarely monotonic with filler content: stiffness may increase while strength and toughness become increasingly sensitive to defects and interfacial integrity. For example, studies on blends of recycled HDPE with milled recycled GFRP (10–40 wt.%) report that increasing filler content generally leads to higher stiffness, while tensile or flexural strength does not necessarily increase and may even decrease at higher filler loadings due to increasing porosity and defect concentration. In particular, materials containing approximately 10 wt.% GFRP typically retains mechanical strength close to that of the unfilled matrix, whereas systems with 30–40 wt.% filler often exhibit greater variability in mechanical performance due to microstructural heterogeneity and defect formation during processing [20]. These results indicate that porosity, defects, and filler dispersion should be treated as key factors governing material performance, rather than as secondary effects [20].

From a micromechanics standpoint, short-fiber and particulate reinforced thermoplastics fail through interacting mechanisms such as fiber pull-out, interfacial debonding, matrix cracking and pore-driven damage, and the apparent “optimum” reinforcement fraction is often the outcome of competing effects: load transfer improves with reinforcement content, but defect sensitivity (e.g., voids, agglomeration, weak interfaces) increases simultaneously. Micro-CT/SEM-based studies explicitly connect pore defects and interfacial damage to failure evolution in short glass fiber reinforced thermoplastic composites, while broader SGFR-thermoplastic analyses underline the decisive role of adhesion and defect initiation sites [21].

Regarding manufacturing routes, polymer recyclates are most commonly reprocessed by extrusion and injection moulding, which are effective but often limited to relatively simple geometries and depend on stable melt behavior [6,9,10,11,12]. In contrast, press moulding (hot pressing) offers a practical route for producing rigid boards from heterogeneous feedstocks; however, property retention becomes highly sensitive to consolidation quality, cooling conditions and defect control. Studies on press forming of fibre-reinforced thermoplastic sheets demonstrate that cooling rate and pressure holding time can significantly influence mechanical performance, supporting the need for clearly defined processing windows in press-formed products [22]. Similar panel-type products in recycled composites (e.g., polymer–fibre systems and WPC-type boards) also underline that morphology and porosity control are central to obtaining robust properties [23,24].

Against this background, the present work investigates an upcycling route for rigid boards manufactured from mechanically recycled pellets derived from post-consumer garment waste, reinforced with ground GFRP waste originating from end-of-life wind turbine blades and consolidated by press moulding. While previous studies have investigated recycled thermoplastics reinforced with conventional short glass fibers or milled GFRP separately, significantly less attention has been devoted to systems combining heterogeneous textile-derived polymer blends with irregular GFRP fragments originating from composite waste streams.

The objective of this study is therefore not only to report the mechanical performance of a specific recycled composite system, but also to analyse the processing–microstructure–property relationships governing such highly heterogeneous materials. In particular, the study focuses on how reinforcement content and consolidation conditions influence flexural strength and Charpy impact behaviour, and how the observed trends can be interpreted in terms of dispersion of the reinforcing phase, interfacial interactions and defect formation during processing.

## 2. Materials and Methods

### 2.1. Materials

**Figure 2:**
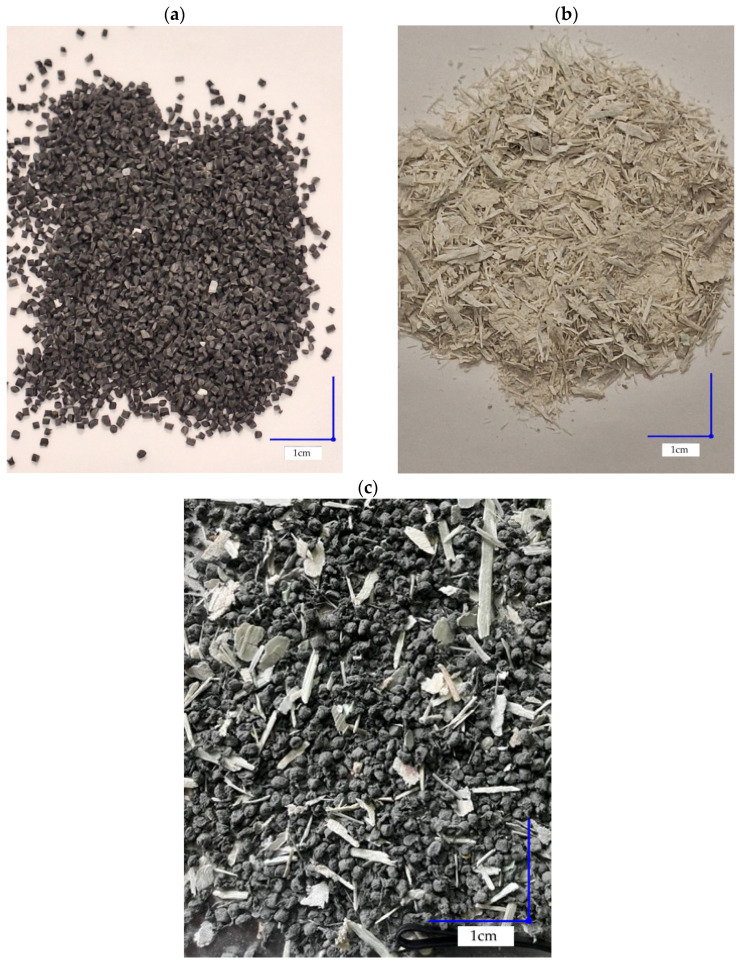
Raw materials used for composite plate fabrication: (**a**) regranulate obtained from mechanically recycled post-consumer textile waste, (**b**) ground GFRP fraction derived from decommissioned wind turbine blades, (**c**) example of the mixed composite feedstock prior to pressing.

The composite plates were manufactured from regranulate obtained through mechanical recycling of post-consumer garment waste supplied by VIVE Textile Recycling Sp. z o.o. (Kielce, Poland). The raw materials used in this study are presented in Figure 2. According to the supplier, the material was classified as an HDPE-based recyclate (polyolefin-rich stream). However, FTIR spectroscopic analysis confirmed that the matrix consisted of a multi-component thermoplastic blend containing high-density polyethylene (HDPE), polyamide (PA6), and polyester (PET), together with various additives and mineral fillers. The reinforcing phase consisted of mechanically ground GFRP waste originating from decommissioned wind turbine blades. The GFRP fraction contained irregular laminate fragments composed of glass fibers embedded in residual cured epoxy resin. Prior to processing, the GFRP material was sieved, and only the fraction passing through a 0.8 mm mesh was used. The diameter of individual glass fibers was not measured directly; however, based on typical e-glass specifications, it is expected to be in the range of approximately 10–20 μm. Typical density values reported in the literature are approximately 0.9–0.95 g/cm^3^ for polyolefin-based recyclates and about 2.55 g/cm^3^ for e-glass fibers.

### 2.2. Composite Preparation

The polymer matrix in the form of regranulate was mechanically mixed with ground GFRP fractions originating from wind turbine blades. The reinforcement was introduced at levels of 10, 20, and 30 wt.%. The GFRP fraction itself was not modified during preprocessing; therefore, the nominal reinforcement content refers to the amount of ground GFRP introduced into the matrix. The compositions of the investigated formulations are summarized in Table 1.

Four formulations were investigated: the unreinforced matrix (P0) and composites containing 10, 20, and 30 wt.% ground GFRP (denoted as P10, P20, and P30). For each formulation, multiple specimens were prepared and tested in order to account for variability associated with the heterogeneous nature of the recycled feedstock.

### 2.3. Microstructural Characterization

Fragment morphology and size distribution were analyzed using optical microscopy and digital image analysis with a VHX-970F digital microscope (Keyence, Osaka, Japan). The observed GFRP fragments corresponded to irregular composite flakes containing glass fibers embedded in cured epoxy resin. The fragment length ranged approximately from 2 to 15 mm, while the fragment thickness remained below 0.8 mm, consistent with the sieving criterion applied during preprocessing. Representative micrographs were used to evaluate fragment morphology and approximate size distribution.

### 2.4. Sample Preparation

Thicker composite plates were produced by hot pressing in a heated steel mold using mixtures of recycled polymer regranulate reinforced with ground GFRP. The preparation procedure consisted of the following steps:

(a) drying of the matrix regranulate,
(b) preparation of mixtures with the required GFRP reinforcement content,
(c) hot pressing in a heated mold,
(d) cooling and demolding of the plates.

**Figure 3:**
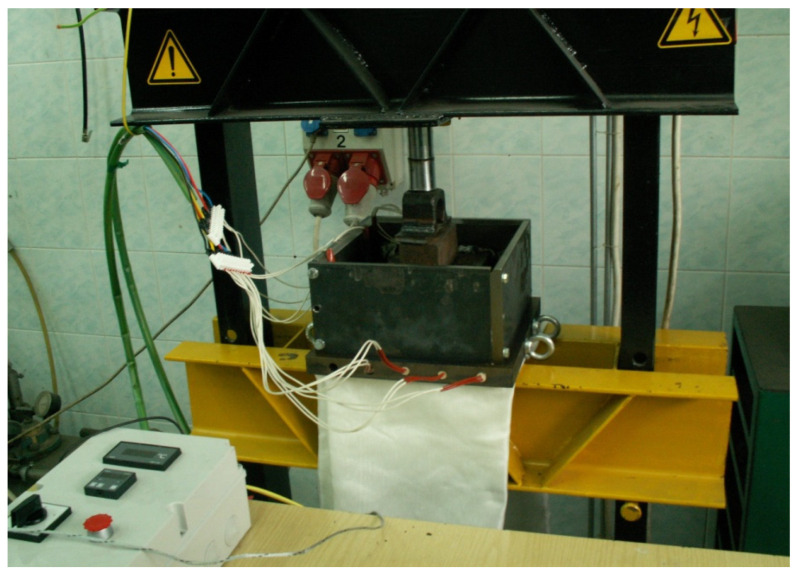
Hydraulic hot press used for fabrication of the recycled composite plates.

**Figure 4:**
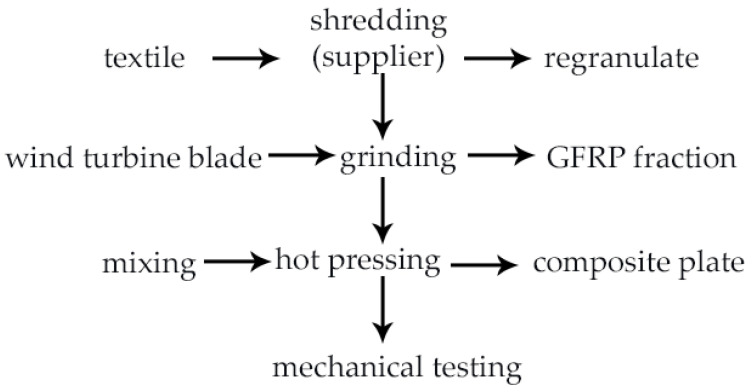
Schematic illustration of the composite plate manufacturing process used in this study: preparation of recycled textile-derived regranulate, preprocessing of ground GFRP waste from wind turbine blades, mechanical mixing of the components, hot pressing, and mechanical testing of the produced composite plates.

Prior to processing, the matrix regranulate was dried at 80 °C for 2 h in order to reduce moisture content. The ground GFRP fraction was used without additional conditioning. Feed mixtures were prepared by adding 10, 20, or 30 wt.% of ground GFRP to the matrix regranulate (Table 1). The components were homogenized in a drum mixer for 15 min to ensure uniform dispersion of the reinforcement phase. The composite plates were fabricated using a hydraulic press equipped with electrically heated platens and a temperature control system (Figure 3). The mold cavity was cleaned before each pressing cycle and no additional release agents were applied. Hot pressing was conducted at a mold temperature of 240 °C under a pressure of 2 MPa, with a heating time of 40 min. The material temperature was measured at the geometric center of the charge before pressing. Due to heat transfer within the packed mixture, the core temperature reached approximately 250 °C during processing. The pressing temperature was selected as a practical processing compromise for the heterogeneous textile-derived polymer matrix. Because the recycled feedstock contained multiple thermoplastic components with different softening and melting behaviour, the temperature had to be sufficiently high to enable matrix softening, flow and consolidation during pressing, while avoiding unnecessarily severe thermal exposure of the recycled material. Therefore, the selected temperature was intended to provide adequate processability of the multicomponent matrix under the applied press-moulding conditions. The resulting plates had approximate dimensions of 200 × 330 mm and a thickness of about 8 mm, with an average mass of approximately 1 kg. Test specimens for mechanical characterization were cut from the plates after cooling to ambient temperature according to the dimensions required for the respective mechanical tests. A schematic illustration of the composite plate manufacturing route is presented in Figure 4. The microstructure of the fabricated composite plates was additionally examined using a digital optical microscope using a VHX-970F digital microscope (Keyence, Osaka, Japan). Surface and cross-section images were recorded at a magnification of 100× to evaluate the dispersion of ground GFRP particles and the presence of defects such as inclusions, voids, and fiber agglomerates.

### 2.5. Mechanical and Structural Testing of Samples

Specimens for mechanical testing were cut from the fabricated plates after cooling to ambient temperature. Due to possible local variations in plate thickness resulting from the hot-pressing process, the cross-sectional dimensions of each specimen (width b and thickness h) were measured individually prior to testing and used in subsequent calculations. The mechanical characterization included a static three-point bending test and an unnotched Charpy impact test, which are commonly used to evaluate the load-bearing capacity and impact resistance of fiber-reinforced polymer composites [25,26]. Due to the heterogeneous nature of the recycled composite system, individual specimens were measured separately. The obtained mechanical properties are reported as mean values ± standard deviation calculated from measurements performed on multiple specimens.

### 2.6. Static Bending Strength Test

The flexural tests were performed using a Zwick Z050 universal testing machine (Zwick/Roell, Ulm, Germany) equipped with a 50 kN load cell. Specimens for the three-point bending test were prepared with a nominal total length of 200 mm. The nominal specimen width was b = 20 mm, while the thickness h corresponded to the actual plate thickness (approximately 8 mm) and was measured individually for each specimen. The support span was L = 160 mm, leaving approximately 20 mm overhang on each side of the specimen. The crosshead speed during the test was set to v = 5 mm/min. The tests were conducted in accordance with ISO 178 [27].

The flexural strength *R*_g_ was calculated according to:

(1) $$R_{g}=\frac{M}{W}=\frac{3\cdot F\cdot l}{2\cdot b\cdot h^{2}}$$

where *M*—bending moment (N·mm), *W*—section modulus (mm^3^), *F*—maximum bending force (N), *l*—distance between supports (mm), *b, h*—width and height (mm) of the sample.

### 2.7. Charpy Impact Test

The impact strength of the plates was determined using the unnotched Charpy method. Rectangular specimens with nominal dimensions of 120 × 15 × 10 mm were used. The tests were performed in accordance with ISO 179 [28]. The use of unnotched specimens was intentional due to the heterogeneous nature of the investigated material (thermoplastic blend reinforced with ground GFRP) and the potential presence of consolidation defects (e.g., porosity, local discontinuities, or glass-fiber agglomerates), which may act as natural crack initiation sites. Consequently, the unnotched configuration was considered more representative of the actual material performance under dynamic loading conditions than the notched variant, which introduces an artificial stress concentrator.

Impact testing was performed using a pendulum impact tester with an energy range of 0–4 J (scale resolution: 0.05 J). Due to the limited plate dimensions, five specimens were prepared from each plate. The pendulum struck the specimen at the midpoint of its width, with the impact direction perpendicular to the plate surface.

(2) $$a_{n}=\frac{A_{n}}{b\cdot h}$$

where *b, h*—width and thickness of the sample, *A_n_*—energy required to break a sample.

### 2.8. FTIR Analysis of Composite Samples

FTIR spectroscopy was employed to identify the main polymeric constituents of the thermoplastic matrix and to support comparison of the chemical composition between the selected plate formulations. Nine representative composite samples corresponding to the tested plates were analyzed. The samples, obtained from the manufacturer, were initially pre-crushed and subsequently ground using a laboratory disc mill (LAB-09-200, Eko-Lab, Brzesko, Poland). The resulting powder was sieved, and the fraction below 0.2 mm was used for FTIR measurements to improve material homogeneity during analysis. The measurements were carried out using a PerkinElmer Spectrum Two spectrometer (PerkinElmer, Waltham, MA, USA) equipped with a UATR (universal ATR) accessory. Spectra were recorded in the range of 4000–500 cm^−1^ at a resolution of 4 cm^−1^, with four scans collected for each sample. During ATR analysis, a constant sample contact force of 73 N was applied. The obtained spectra were baseline-corrected and normalized prior to interpretation. Identification of individual components was based on characteristic absorption bands of polyolefins (HDPE/PE), polyamide (PA6), and polyester (PET). The results were used to provide indicative information on the composition of the thermoplastic matrix (Table 2).

## 3. Results and Discussion

### 3.1. Chemical Composition Analysis and Microstructural Characterisation of the Plates Obtained

**Figure 5:**
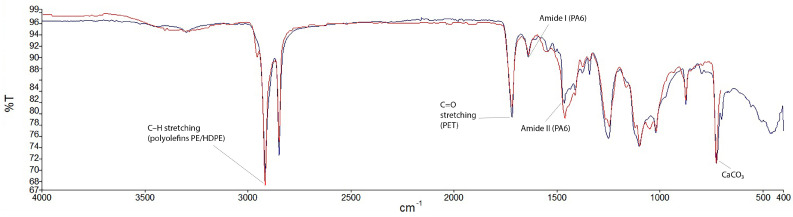
ATR-FTIR spectrum of the investigated composite sample (P1) with identified characteristic absorption bands. The blue curve represents the measured spectrum, while the red curve corresponds to the fitted/reference-matched spectrum used for spectral interpretation.

The ATR-FTIR spectrum of the investigated composite sample is presented in Figure 5. The spectral analysis confirms that the thermoplastic matrix used for plate fabrication is a multi-component polymer blend containing mineral additives. Characteristic absorption bands corresponding to C–H stretching vibrations of aliphatic polyolefins are observed at approximately 2916 and 2848 cm^−1^. Features associated with polyester components (PET) are identified by the strong C=O stretching band at 1715–1730 cm^−1^ and C–O–C vibrations between 1100 and 1240 cm^−1^ [29]. The presence of polyamide (PA6) is confirmed by characteristic amide I (1630–1650 cm^−1^) and amide II (1530–1560 cm^−1^) bands [29].

Additional bands at approximately 1410–1470 cm^−1^, ~870 cm^−1^, and ~710 cm^−1^ indicate the presence of calcium carbonate (CaCO_3_), likely used as a mineral filler. A broad absorption band in the 3200–3600 cm^−1^ region suggests hydroxyl-containing components or absorbed moisture. The coexistence of polyolefin and polar polymers (PET, PA6) together with mineral fillers indicates a heterogeneous, multi-component matrix structure.

The estimated composition of the thermoplastic blend, obtained by spectral fitting using reference libraries, is summarized in Table 2. These values should be treated as semi-quantitative estimates rather than absolute mass fractions. The multi-component nature of the matrix may influence phase compatibility and interfacial interactions, which can affect the mechanical performance of the composites discussed in the following sections.

**Figure 6:**
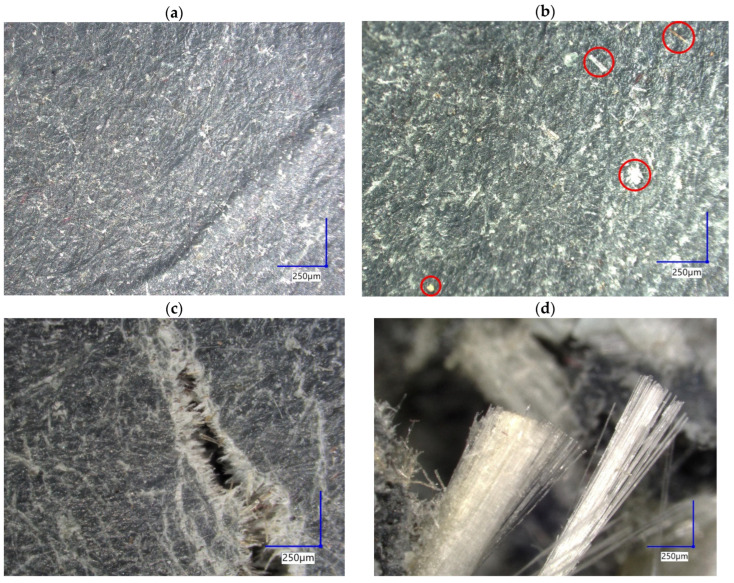
Optical micrographs of hot-pressed composite plates containing ground GFRP fraction (VHX-970F digital microscope, Keyence, Osaka, Japan), 100× magnification; scale bar: 250 μm. (**a**) P1 (30 wt.% GFRP)—relatively homogeneous surface structure; (**b**) P2 (30 wt.% GFRP)—surface with local inclusions; (**c**) P3 (30 wt.% GFRP)—cross-section showing fibrous structure and local porosity; (**d**) P4 (20 wt.% GFRP)—cross-section with visible GFRP fiber bundles. Red circles indicate selected local defects and heterogeneities discussed in the text.

Figure 6a–d present representative optical micrographs (100× magnification) of plates fabricated by hot pressing. The observations reveal a pronounced microstructural heterogeneity typical of multi-component recyclates reinforced with a ground GFRP fraction. Surface images (Figure 6a,b) show areas of relatively homogeneous matrix interspersed with visible local agglomerates (e.g., mineral fillers) and occasional inclusions (Figure 6b). Cross-sectional images (Figure 6c,d) show the presence of glass-fiber-rich zones, fiber bundles, and local porosity and discontinuities, which may suggest incomplete dispersion of the GFRP fraction and non-uniform consolidation during pressing. It should be noted that the presented images correspond to samples with different glass fiber contents (30 wt.% GFRP for P1–P3 and 20 wt.% GFRP for P4), highlighting the local character of the observed features, which result from dispersion quality and consolidation conditions. Such microstructural characteristics may increase the material’s sensitivity to defects (e.g., pores, weak interfacial regions, agglomerates) and may therefore contribute to the observed scatter in mechanical results and the absence of a monotonic relationship between mechanical properties and glass fiber content in both flexural and impact tests.

### 3.2. Bending Test Results

The results of the three-point bending tests are summarized in Table 3. The table presents the mean flexural strength values and corresponding standard deviations for composites containing 0, 10, 20, and 30 wt.% of ground GFRP.

The averaged flexural strength values obtained for different reinforcement levels are presented in Table 3. The reference material without GFRP exhibited a flexural strength of 13.10 ± 1.21 MPa. For the composites containing ground GFRP, the mean values were 11.90 ± 0.27 MPa (10 wt.% GFRP), 10.35 ± 1.40 MPa (20 wt.% GFRP), and 11.37 ± 2.14 MPa (30 wt.% GFRP). No monotonic relationship between reinforcement content and flexural strength was observed, suggesting that the reinforcing effect of ground GFRP may be influenced not only by its content but also by dispersion quality and interfacial interactions within the composite. The lowest average value occurred for the composite containing 20 wt.% GFRP, whereas the 30 wt.% GFRP material exhibited slightly higher strength but also a noticeably larger scatter of results. This behavior may be attributed to the competition between improved load transfer provided by the glass fibers and the increased sensitivity of the material to defects and microstructural heterogeneity. In particular, the obtained results may be influenced by several factors:

(a) reinforcement dispersion and interfacial adhesion—local fiber agglomeration and weak interfacial bonding may promote stress concentration and earlier crack initiation;
(b) consolidation quality during pressing—higher GFRP content may hinder densification of the charge and promote the formation of pores and voids;
(c) the multi-component nature of the polymer matrix—the heterogeneous composition of the matrix (different polymers and additives) may locally alter matrix properties and affect reinforcement efficiency.

**Figure 7:**
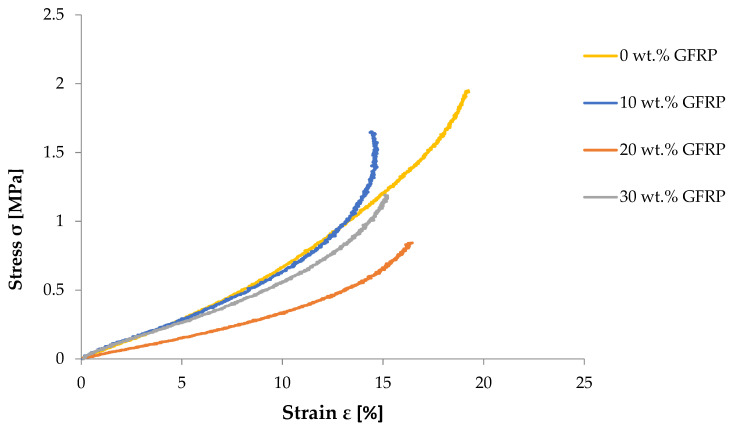
Representative flexural stress–strain curves from three-point bending tests of recycled polymer blend plates reinforced with different contents of ground GFRP (0, 10, 20 and 30 wt.%).

Representative flexural stress–strain curves obtained in the three-point bending test are shown in Figure 7.

### 3.3. Impact Test Results

Based on the results of the Charpy impact test, the impact strength was calculated according to Equation (2). The average Charpy impact strength values obtained for each composition are summarized in Table 4. The reported values represent the mean and standard deviation calculated from individual measurements (N = 9 for 10, 20, and 30 wt.% GFRP; N = 3 for the reference sample).

The similar impact strength values obtained for different ground GFRP contents (10, 20, and 30 wt.%) do not indicate a monotonic relationship between reinforcement content and resistance to dynamic loading. The observed variations in a_n_ fall within the range of experimental scatter, suggesting that, in the investigated system, the reinforcement fraction is not the sole factor determining impact performance. In short-fiber composites, impact strength depends to a large extent on fiber length and orientation, interfacial adhesion quality, and microstructural homogeneity [25,26,29].

1. Saturation Threshold and Limited Reinforcement Efficiency in Impact

In short-fiber systems, including heterogeneous recycled composites such as the one investigated here, increasing fiber content does not necessarily result in higher impact strength. Beyond a certain reinforcement level, the energy absorption mechanism may shift from one dominated by fiber pull-out and controlled debonding to one governed by crack initiation and propagation in regions of stress concentration. Consequently, stabilization—or even a decrease—of impact strength may be observed at higher fiber contents [25,30].

2. Dispersion and Interfacial Adhesion at the Fiber–Matrix Boundary

Impact strength is particularly sensitive to the efficiency of stress transfer at the phase boundary. Non-uniform fiber dispersion, the presence of agglomerates, and insufficient interfacial adhesion may limit the activation of energy absorption mechanisms, such as fiber pull-out or controlled microcracking with interfacial debonding. Under such conditions, increasing the GFRP content does not necessarily translate into a proportional increase in impact strength a_n_ [29,30].

3. Matrix Properties and Heterogeneity

The variable, multi-component nature of the polymer matrix (different polymers and additives, e.g., CaCO_3_ and HEC) may significantly influence impact performance. Local variations in ductility and plastic deformability can compensate for potential benefits resulting from increased GFRP content, leading to only limited changes in a_n_.

4. Morphology of the Ground GFRP Reinforcement and Consolidation Defects

In contrast to conventional short glass fiber-reinforced thermoplastic composites (SGFR-TP) based on virgin matrices, ground GFRP contains fibers of varying lengths as well as residual cured resin, which hinders the formation of a uniform microstructure. Moreover, at higher GFRP contents, the tendency for pore formation and local stress concentration may increase, which limits impact performance and leads to a “flattening” of the trend with increasing reinforcement content [30].

An additional factor limiting the reinforcement efficiency of the composite is the degradation of fiber morphology resulting from the mechanical grinding of wind turbine blades. During comminution, significant shortening of the glass fibers occurs, along with partial surface damage. Consequently, the effective fiber length may approach or even fall below the critical length, thereby limiting full stress transfer from the matrix to the reinforcing phase [31]. Under such conditions, the fibers do not function as classical continuous or semi-continuous reinforcement, but instead exhibit behavior closer to that of an irregular fibrous–particulate filler. It should also be noted that the ground GFRP fraction contains not only glass fibers, but also fragments of cured epoxy resin and potential surface contaminants. This results in the formation of a hybrid system in which fibrous and particulate elements of varying stiffness and interfacial adhesion coexist. Such morphology promotes local stress concentrations and microcrack initiation, particularly at higher GFRP contents. Literature on the recycling of GFRP composites indicates that structural fiber degradation and interfacial weakening are among the main factors limiting mechanical property retention in secondary materials [19,30,32,33,34]. Consequently, the observed lack of monotonic increase in strength and the stabilization of impact performance at 20–30 wt.% GFRP may result from the superposition of two opposing effects: on the one hand, an increased contribution of the stiff reinforcing phase; on the other hand, enhanced sensitivity of the material to consolidation defects, non-uniform dispersion, and limited stress transfer efficiency. This mechanism is consistent with observations reported for short-fiber composites, in which reinforcement efficiency is governed by effective fiber length, interfacial adhesion quality, and porosity control [25,30,35].

To relate the obtained results to conventional short glass fiber-reinforced thermoplastic systems based on virgin matrices, it should be emphasized that in SGFR-TP materials, impact performance is strongly determined by effective fiber length, fiber orientation, and interfacial adhesion quality [25,29,30,35]. The literature also indicates that increasing fiber content above approximately 30 wt.% does not necessarily lead to further improvement in impact strength; in many cases, stabilization or even deterioration is observed, associated with increased brittleness and microstructural defect concentration [25,30].

## 4. Conclusions

The present study investigated the mechanical performance of recycled multicomponent textile polymer blends reinforced with ground GFRP originating from wind turbine blades and consolidated by hot pressing. The obtained results show behaviour consistent with that reported for heterogeneous recycled thermoplastic composites containing glass-fiber-based reinforcement, in which increasing reinforcement content does not necessarily lead to monotonic improvement in mechanical performance because of the combined effects of dispersion quality, interfacial limitations, and processing-related defects. Based on the obtained results, the following conclusions can be drawn:

(a) The incorporation of ground GFRP (10–30 wt.%) into the recycled polymer matrix did not result in a monotonic improvement in flexural or impact properties compared with the unreinforced material.
(b) The highest average flexural strength was observed for the reference material without reinforcement, indicating that the addition of ground GFRP did not necessarily enhance the load-bearing capacity of the investigated recycled system.
(c) Charpy impact strength values remained within a relatively narrow range (7.3–8.46 kJ/m^2^), showing no clear dependence on reinforcement content.
(d) The mechanical performance of the investigated composites appears to be influenced primarily by microstructural heterogeneity, fiber morphology, and interfacial interactions rather than by reinforcement fraction alone.
(e) The results indicate that ground GFRP recovered from wind turbine blades can be incorporated into recycled polymer blends; however, the effectiveness of reinforcement may be limited by fiber fragmentation, dispersion quality, and consolidation-related defects.
(f) From a practical perspective, the investigated system demonstrates the potential for producing rigid boards from mixed textile-derived polymer recyclates and mechanically ground GFRP waste, although further optimization is required to improve reinforcement efficiency and property consistency.

## Figures and Tables

**Table 1:** Composition of the investigated composite formulations.

Sample ID	Matrix (wt.%)	Ground GFRP (wt%)
P0	100	0
P10	90	10
P20	80	20
P30	70	30

**Table 2:** Semi-quantitative composition of the thermoplastic matrix regranulate estimated based on ATR-FTIR spectral fitting [%].

No.	Sample Designation	Polyamid	Thermoplastic Polyester	PE/HDPE	Fillers **
1.	P0 *	25.22	21.81	16.48	36.49
2.	P1	29.15	20.89	13.98	35.98
3.	P2	28.00	22.00	17.99	32.01
4.	P3	25.80	20.45	16.60	37.15
5.	P4	26.89	21.99	18.70	32.42
6.	P5	20.50	20.17	18.80	40.53
7.	P6	24.70	21.79	12.63	40.88
8.	P7	25.64	19.86	15.67	38.83
9.	P8	22.75	26.45	19.12	31.68
10.	P9	23.56	22.69	14.83	38.92

** Fillers—total fraction assigned by the ATR-FTIR fitting procedure to components other than PA6, PET, and PE/HDPE (mainly CaCO_3_-containing fractions and components with hydroxyl functional groups, e.g., HEC). The reported values are semi-quantitative estimates derived from library-based spectral fitting and should not be interpreted as exact compositional values or absolute mass fractions. Row totals are approximately 100% (differences result solely from rounding). * P0—reference sample (0 wt.% GFRP), i.e., thermoplastic matrix without ground GFRP addition, analyzed by ATR-FTIR under the same conditions as the other samples.

**Table 3:** Average flexural strength values obtained in the three-point bending test for composites containing different amounts of ground GFRP (mean ± SD).

GFRP Content (wt.%)	Three-Point Bending			
	Flexural Strength R_g_ (MPa)	SD		N
0	13.10	1.21		3
10	11.90	0.27		9
20	10.35	1.4		9
30	11.37	2.14		9

**Table 4:** Average Charpy impact strength values obtained for composites with different ground GFRP contents (mean ± SD).

Charpy Impact Test			
Ground GFRP Content (wt.%)	Impact Strength a_n_ [kJ/m^2^]	SD	N
0	7.30	0.20	3
10	8.03	0.74	9
20	8.46	0.55	9
30	8.10	0.46	9

## Data Availability

The original contributions presented in this study are included in the article. Further inquiries can be directed to the corresponding author.
